# Retraction: Prostaglandin E1 protects cardiomyocytes against hypoxia-reperfusion induced injury via the miR-21-5p/FASLG axis

**DOI:** 10.1042/BSR20190597_RET

**Published:** 2026-08-07

**Authors:** Mingxiang Tang, Hongwei Pan, Zhaofen Zheng, Yin Guo, Jianqiang Peng, Jun Yang, Yangping Luo, Jin He, Sulan Yan, Peng Wang, Yi Zhang, Yulu Zhou

**Affiliations:** 1 Department of Cardiology, Hunan Provincial People's Hospital & First Affiliated Hospital of Hunan Normal University, Changsha, Hunan, China; 2 Department of Pharmacy, The third Xiangya Hospital of Central South University, Changsha, Hunan, China

**Keywords:** apoptosis, cardiomyocytes, hypoxia/reoxygenation, prostaglandin E1

This article is being retracted from *Bioscience Reports* at the request of the Editor-in-Chief and the Editorial Board. This follows an internal investigation, which identified various points of concern within the article. Specifically, these include the following instances of high similarity:

- Between the Figure 1B GAPDH blots (lanes 1-4) of this article and the Figure 1B GAPDH blots from Li et al. 2019 [RETRACTED] (DOI: 10.1186/s11658-019-0139-z)
- Between the bottom two quadrants of the Figure 2C scatterplot 1 μM and 10 μM panels
- Between the middle region of the Figure 7A control panel and the 7D 6H/6R + over+FASLG + miR-21-5p mimics panel
- Between the Figure 7C GAPDH blots (lanes 2-4) of this article and the Figure 11B GAPDH blots (lanes 1-3) of Chen et al. 2021 (DOI: 10.18632/aging.202832)

The authors have been contacted with regard to the retraction and have not responded to the Journal's queries or to the concerns. Given the extent of the issues raised, the Editorial Board stands by the decision to retract the article.

